# Notes and Notices

**Published:** 1898-12

**Authors:** 


### NOTES AND NOTICES.

Speer's Port Grape Wine is the most pleasing and comforting beverage that can be given an invalid. Ask your doctor.

Brookside Sanitarium for mental cases, is a private institution under State supervision. Located on the mountain side, with seventy acres of lawn, forest, orchard, and garden. A fine new mansion erected for the purpose. Large, airy rooms, heated by hot water, and lighted by electricity; all modern improvements. Driving as a means of entertainment and recreation—a specialty. The very best food, care, and treatment. Rates from $12 per week upward, according to the needs of the case and accommodations desired.

New York and Washington Ladies in delicate condition derive great benefit from Speer's Port Wine. Also excellent for the aged and infirm and convalescents.

Marchand's Hydrozone.—When you cannot procure from your jobber Marchand's medicinal preparations in their original, unbroken packages, viz.: Peroxide of Hydrogen (medicinal), Glycozone, Hydrozone and Eye Balsam, please address your order to either Messrs. Schieffelin & Co., 170 William Street, N. Y., or myself, when orders will be filled promptly.

Yours truly,

Charles Marchand,

New York, 57-59 Prince Street, cor. New Elm Street, October 17th, 1898.

See a lot of men in another column treading grapes at Do Sexio, Portugal, for wine. Read all about it and about Speer's N. J. method and what it is particularly good for.

Crew Got Fat on Arsenic.—The German bark "Zion," which arrived at this port Sunday from Fowey, England, brought a rather peculiar cargo. It consisted of

1,800 casks of china clay, but in addition there were on board 300 casks of Arsenic. This part of the cargo had a remarkable effect on the crew.

The fact that Arsenic as well as Strychnine helps the formation of adipose tissue when taken into the human system in minute particles is well known, and both drugs have become favorite tonics for convalescents.

On board the "Zion" the men slept very near the large array of barrels containing the drug. They were stored in the hold, near the forecastle, and partially exposed to the rays of the sun, which streamed in through the open hatch. When only about a week out from port one of the crew mentioned to his messmates that a peculiar and indescribable odor was coming from the casks containing the drug. It was not long after their attention had been called to it that they all noticed the same thing, and strange to say, noticed it all the more forcibly a week later. Several of the German tars became aware of the fact that they were filling out their clothes to a much greater extent than when they shipped. Many others, as days went on, became abnormally stout, in vast contrast to the former slim appearance which many of them presented before the land was left. One man gained, it is said, twenty-five pounds. Others were affected to a less extent. But the aggregate extra weight put on by the entire crew was a little less than four hundred pounds.

Several of the sailors are known here, and they are said to be scarcely recognizable when contrasted with the old days. The entire sudden taking on of avoirdupois is attributed to vapor, which, generated by the action of the sun on the casks, was inhaled by the seamen as they slept, and acted in precisely the same manner which it does when given as a tonic in a prescription. Captain Hammes, who slept aft in the vessel, entirely removed from the Arsenic, does not show any effect of the inhalation.

The "Zion," after discharging her cargo, will load oil at Point Breeze for London, when it is supposed the seamen will return to their normal condition.—Philadelphia *Times*, November 1st, 1898.

Surgery in the Middle Ages.—Dr. Robert Fletcher in his *Anatomy of Art*, and Dr. Luigi Sambon having shown conclusively that Greeks and Romans must have had a good acquaintance with surgery, it seems strange that in the mediæval European period there was dense ignorance and no skill in amputation. Sword and lance wounds were necessarily of constant occurrence then, and the treatment was merciless. We have shown before how there was among primitive people a fair acquaintance with surgery, and even a knowledge of the refinements of it as in plastic operations. The discovery of a manuscript of the eleventh century shows us conclusively that among the Arabs and in Syria at the time of the first crusades, there was a fair knowledge of surgery, and that the Syrians held in poor estimation the Frank doctor. Osama tells how a knight was suffering from an abscess of the thigh, and a woman from consumption. The Frank physician had the knight's leg put in a block, and it was hacked off with a sword. The woman was treated by having her hair cut and a cross cut into her skull. The knight died at once, and so did the woman. Then the chronicler says the Syrian doctor who had been called in left disgusted, having learned more about Frankish medicine than he had ever known before.—*Scientific American*, August 1st, 1896.

